# The tick biocontrol agent *Metarhizium brunneum* (= *M*. *anisopliae*) (strain F52) does not reduce non-target arthropods

**DOI:** 10.1371/journal.pone.0187675

**Published:** 2017-11-20

**Authors:** Ilya R. Fischhoff, Felicia Keesing, Richard S. Ostfeld

**Affiliations:** 1 Cary Institute of Ecosystem Studies, Sharon Turnpike, Millbrook, NY, United States of America; 2 Bard College, Annandale-on-Hudson, New York, United States of America; University of Kentucky College of Medicine, UNITED STATES

## Abstract

Previous studies have found that Met52®, which contains the entomopathogenic fungus *Metarhizium brunneum*, is effective in reducing the abundance of *Ixodes scapularis*, the tick vector for the bacterium causing Lyme disease and for other tick-borne pathogens. Given widespread interest in effective, safe methods for controlling ticks, Met52 has the potential to be used at increasing scales. The non-target impacts of Met52, as applied for tick control, have not yet been assessed. A Before-After-Control-Impact experiment was conducted to assess the effects of Met52 on non-target arthropods in lawn and forest habitats typical of residential yards. Ground-dwelling arthropods were collected using bulk sampling of soil and litter, and pitfall sampling. Arthropods were sampled once before and twice after treatment of plots with either Met52 or water (control). Multivariate general linear models were used to jointly model the abundance of arthropod orders. For each sampling method and post-spray sampling occasion, Akaike Information Criterion values were used to compare the fits of two alternative models: one that included effects of period (before vs. after spray), habitat (lawn vs. forest), and treatment (Met52 vs. control), versus a nested null model that included effects of period, and habitat, but no treatment effect. The null model was consistently better supported by the data. Significant effects were found of period and habitat but not treatment. Retrospective power analysis indicated the study had 80% power to detect a 50% reduction in arthropod abundance, as measured by bulk samples taken before versus one week after treatment. The deployment of Met52 in suburban settings is unlikely to cause meaningful reductions in the abundance of non-target arthropods.

## Introduction

An estimated 300,000 Lyme disease cases occur annually in the United States, making it the country’s most common vector-borne disease [1]. Without treatment, Lyme disease can cause severe joint, heart, and neurological symptoms. The blacklegged tick *Ixodes scapularis* transmits the bacterium *Borrelia burgdorferi*, which causes Lyme disease. *I*. *scapularis* also transmits the bacterium that causes anaplasmosis, the protozoan that causes babesiosis, and Powassan virus. The geographic range of Lyme disease is expanding in North America [2]. Health officials and the public seek solutions to reduce the incidence of tick-borne diseases (TBD) cost-effectively and safely.

Diverse strategies have been employed to reduce TBD, including approaches focused on people, wildlife, and ticks [3]. A human vaccine against *B*. *burgdorferi* was available in the late 1990s and early 2000s, but was withdrawn from the market following low demand and concerns about efficacy and potential side effects [4]. The few randomized, controlled studies of educational interventions indicated people’s capacity to adopt tick prevention behaviors, yet these interventions did not reduce TBD [5].

Wildlife-focused approaches include removing, vaccinating, and protecting hosts against ticks. Evidence from experimental studies does not support reducing or removing deer, the primary hosts for adult ticks, as a strategy, except in isolated areas [6,7]. Vaccination of white-footed mice *Peromyscus leucopus* against *B*. *burgdorferi* via oral baits reduced infection prevalence in ticks within 3 years; however, this vaccine is not available commercially [8]. Topical application of acaricides on deer, via bait stations, reduced the density of infected ticks [9]; however, this reduction was less than 10% when bait stations were deployed at a lower density feasible for land managers [10]. Application of acaricides on small mammals via bait boxes also reduced the density of infected ticks in residential yards [9].

Tick control efforts have focused on residential yards, where most tick encounters are thought to occur in the eastern and central United States [11–13]. Tick density has been reduced by yard treatments with chemicals [14]. However, a randomized, controlled trial with bifenthrin found that reduction in yard ticks was not accompanied by reduction in TBD diagnoses in residents [15]. One possible explanation for this result is that participants in the study may have encountered ticks outside their yards, or in parts of their yards for which bifenthrin is contraindicated and therefore were unsprayed (e.g., vegetable gardens) [15]. Bifenthrin poses risks to non-target arthropods [16]. For example, Coleoptera, Hymenoptera, and Collembola were several times less abundant in forest plots, one week following treatment with bifenthrin for blacklegged tick control, compared to their abundances in reference plots [17]. Other chemical acaricides, such as chlorpyrifos, pose human health risks [18]. Only 47% of Connecticut survey respondents were willing to spray chemicals for tick control, safety being the most frequently cited reason for those unwilling to use chemicals [19]. Among Swiss and Canadian survey respondents, use of chemical acaricides was acceptable for fewer than 30%, whereas biocontrol was acceptable to over 75% [20].

Given public concerns about chemicals, and continued increases in Lyme and other TBD, researchers have investigated the tick control potential of natural products and biocontrol agents. Nootkatone, extracted from Alaska yellow cedar *Chamaecyparis nootkatensis*, controlled ticks in field trials; however, nootkatone must be developed to be cost-effective and have longer-term efficacy [21]. Certain nematodes kill ticks but cannot complete their life cycle in them, leading to short-lived effects [22]. The parasitic wasp *Ixodiphagus hookeri*, native to Europe and introduced in the United States, has been evaluated for biocontrol, but it persists only at extremely high tick densities [23].

Among tick biocontrol agents, entomopathogenic fungi appear to have the greatest potential [24]. *Metarhizium brunneum* F52, previously classified as *M*. *anisopliae* [25], has been incorporated into a commercial product, Met52 (Novozymes Biological, Franklinton, NC, USA). The F52 strain was first cultivated from the codling moth *Cydia pomonella* in Austria [26]. Field tests with Met52 resulted in reductions in *I*. *scapularis* comparable to those achieved with bifenthrin [21].

The Tick Project (www.tickproject.org) is a 5-year study (2016–2020) to determine whether controlling ticks at the neighborhood scale reduces TBD. The Tick Project is evaluating two methods of tick control, applied separately or together in yards: 1) Met52 and 2) bait boxes that apply the acaricide fipronil to small mammals. These two methods were selected based on their commercial availability, efficacy, and safety.

In assessing Met52, it is important to evaluate not only its efficacy in reducing TBD but also its non-target impacts. Previous studies on the non-target impacts of Met52 have been in the lab or in agriculture. For terrestrial vertebrates, Met52 has been found safe, based on tests with rats and bobwhite quail *Colinus virginianus* [27]. The Environmental Protection Agency further concluded that terrestrial uses of Met52 do not adversely affect aquatic animals based on tests with rainbow trout *Oncorhynchus mykiss* and *Daphnia major*. Among terrestrial arthropods, no effect of F52 was detected in lab tests with parasitic wasps *Nasonia vitripennis*, honeybees *Apis melifera*, lady beetles *Hippodamia convergens*, lacewings *Chrysoperla carnea*, or earthworms *Eisenia fetida* [27]. Exposure to *M*. *brunneum* BIPESCO 5 (= F52) resulted in increased mortality in the collembolan *Folsomia fimetari* [28] and the predatory bug *Orius majusculus* ([29]. In a greenhouse, Met52 caused mortality in beneficial predators: rove beetles *Dalotia coriaria* and mites *Stratiolaelaps scimitus* and *Gaeolaelaps gillespiei* [30].

In Hungarian maize fields, application of BIPESCO 5 (= F52) resulted in no significant effect on non-target species composition [31]. Following F52 treatment, infection with F52 was observed in non-target Coleoptera in Danish lucerne fields [32], and in Coleoptera and Hemiptera, but not Pscocoptera, in a Danish fir plantation [33]. The non-target effects of other *Metarhizium* strains have also been field-tested. In a Spanish olive orchard, ant abundance was higher in the *Metarhizium* plot than the control plot [34]. No effects of *Metarhizium* were found on arthropod presence in savanna woodland in Niger [35], ant diversity in Kenyan savanna [36], soil arthropod abundance in a German vineyard [37], or arthropod predator abundance in Chinese grasslands [38]. The potential for Met52 to have non-target effects is suggested by its virulence against diverse targets: Coleoptera [39], Diptera [40], Hemiptera [41], Hymenoptera [42], Orthoptera [38], and Thysanoptera [43].

The non-target effects of Met52, as applied against ticks in a suburban landscape, have not been previously studied in the field. Using a Before-After-Control Impact (BACI) design, we compared the abundance of ground-dwelling arthropods in treatment and control plots, before and after spray with Met52 on the treatment plots or water on the control plots.

## Materials and methods

### Experiment location and study design

Experimental locations were on the grounds of the Cary Institute of Ecosystem Studies (CIES) (Millbrook, NY, U.S.A). Each of the 13 locations comprised a pair of adjacent 8m x 8m plots. Based on a coin flip, we designated one plot in each pair for spray with Met52 and one plot for spray with an equal volume of water. Lawn and forest were both included in each experimental location because these are two of the main habitat types in residential yards within the Lyme disease endemic zone. Each 8m x 8m plot comprised a 4m x 8m area of regularly mown lawn, next to a 4m x 8m area of forest. To minimize drift of Met52 into control plots, Met52 and control plots were separated by 3 meters. Each location was at least 20 meters from other locations.

Pairs of plots at thirteen locations were sprayed once over the period 29 June 2016 to 15 July 2016. We sprayed each plot with a hydraulic sprayer at a pressure of 200 pounds-per-square-inch (1,379 Kilopascals). We applied Met52 at the dosage recommended to control ticks [44]. The product label indicates to apply Met52 EC^®^ against ticks at a rate of 2 to 3 ounces of concentrate, diluted in a minimum of 4 gallons water, per 1000 square feet (93 square meters) [44]. We applied 3 oz of Met52, in 11.5 gallons of water, per 1000 square feet. A greater volume of water was used, compared to the minimum required, to ensure sufficient volume to cover the surfaces of vegetation to a height of 90 cm. To minimize cross-contamination, the sprayer was triple-rinsed with water in between use with Met52 and with water.

### Non-target arthropod sampling

Bulk and pitfall sampling were used to collect ground-dwelling arthropods, which were expected to have greatest Met52 exposure.

#### Bulk samples

Peak Met52 impacts occur within days to weeks, depending on target taxa and environmental conditions [44]. Given this range of potential peak times, we sampled at two post-treatment intervals. We collected bulk soil, litter, and lawn samples within 1 week prior to treatment, at 1 week post-treatment, and 3 weeks post-treatment.

For each sampling occasion, two samples were taken in the lawn half of each plot. Each lawn sample included both grass and underlying soil to a depth of 5 cm, with diameter 10 cm. The litter and soil portion of each lawn sample was extracted and processed together, as it was not practical to separate the two. To account for potential edge effects, we stratified sampling by distance to the lawn-forest border. One lawn sample was taken from the center of one of eight 1m x 2m quadrats along the lawn-forest edge, while the other sample was taken from one of eight 1m x 2m quadrats away from the forest edge. We chose quadrats randomly, sampling each quadrat no more than once.

For each sampling occasion, we also selected two sample locations in the forest half of each plot, using the same protocol as for lawn. At each sample location, we took a litter sample 10 cm in diameter, and a sample of soil (underneath the litter) 10 cm in diameter and 5 cm in depth. Lawn and forest soil samples were taken using a turf cutter (Miltona Turf Tools, Lino Lakes, MN, USA). Litter samples were taken using a bread knife to cut around the band of a springform pan. To minimize cross-contamination, we used separate sampling equipment for Met52 and control plots and wore disposable booties when entering Met52 plots post-spray. We processed the litter and soil separately from each forest sampling location.

Samples were stored at 4°C for no more than 72 hours prior to being placed under a 15 Watt bulb for 48 hours in a Berlese funnel over a jar holding 70% ethanol. The bulb was installed in a clamp light, placed on an 8-quart funnel (Behrens, Winona, MN, USA), held up with a bucket. We wrapped each sample loosely in coarse (grade 10) cheesecloth and then placed it on top of window screening and 0.5 inch wire mesh in the funnel. The cheesecloth and window screening served to reduce dirt falling down the funnel into the ethanol. The circular piece of window screen material was placed on the center of the wire mesh and extended to two inches from the walls of the funnel, facilitating macroinvertebrates moving through the wire mesh and down the funnel to the collection jar.

Prior to sorting, we distributed the contents of each samples evenly onto a 90 mm circle of 41 micron nylon mesh (Elko Filtering, Miami, FL USA) by pouring the sample through a 90mm vacuum filter (Fisher, Pittsburgh, PA, USA). The filtration process retained on the filter any organisms greater than 41 microns in size. After sieving, we placed the filter on a petri dish. Due to high numbers of Acari and Collembola, 15% of each sample was counted for these orders. Subsampling was performed using a gridded sticker adhered to the bottom of the petri dish. Grid cells were randomly selected, equal to 15% of the filtered area of the mesh. Acari and Collembola were counted in the same set of grid cells in each sample. The total numbers of Acari and Collembola in each sample were estimated by extrapolation: estimated total = (100 / 15) X (count of subsample). Within Acari, separate tallies were kept for mites and for *I*. *scapularis*, the target taxa for Met52. Only one *I*. *scapularis* was found, and analyses for Acari included mites only. We identified to order and counted all other specimens [45,46]. For all orders, we counted larvae together with adults. Sorters did not know the treatment of each sample.

#### Pitfall samples

We used pitfalls to sample macroarthropods at seven of the 13 locations, before and 1 week after spraying. We conducted pitfall sampling at a subset of sites due to time constraints. At 3 locations, an additional sample was taken 5 weeks post-spray. Pitfalls were 16-oz deli containers (10 cm diameter, 5 cm depth), buried to be flush with the soil surface, and covered by a 30 cm square wooden coverboard suspended 2 cm over the ground by lawn pegs. At each sampling occasion, pitfalls were filled with 60 ml of 70% ethanol and left open for 24–48 hours (times varied due to logistical constraints). We deployed pitfalls in fixed locations. Each habitat (lawn, forest) had two pitfall locations, with locations stratified by distance to the lawn-forest border as with the bulk samples. We placed pitfall traps in different quadrats from those used for bulk sampling. Prior to sorting, we sieved samples with a 500 micron mesh. We then sorted samples to order, counting every individual.

Fieldwork was conducted with permission of the CIES. No protected species were sampled.

### Data analysis

#### Data pooling

We pooled abundance data for each order within each plot, sampling occasion, habitat, and sample type (bulk versus pitfall). Bulk samples included 467 samples (156 lawn, 156 forest soil, and 155 forest litter samples, 1 litter sample being lost). We pooled these into 156 pooled samples (3 sampling occasions at 13 locations, each location containing 2 plots, each plot with 1 pooled lawn and 1 pooled forest sample). The pitfall samples included 129 samples (62 forest and 67 lawn, 7 samples being too dirty to sort). Pitfall samples were pooled into 68 samples.

#### Modeling abundance of arthropod taxa

We analyzed the data using multivariate generalized linear models (GLMs), with function “manyglm” in R package “mvabund” [47,48]. We used R version R 3.4.0. Manyglm jointly predicts abundance across multiple taxa. Variance in abundance was greater than the mean for most orders. Therefore, abundance of order *j* in sample *i* was modeled as negative binomial: *Y*_*ij*_ ~ *NB*(*μ*_*j*_, *Φj*).

The effect of treatment was tested by comparing the fit of a model that included treatment as a predictor, versus a null model that did not include treatment. The null model for abundance of order *j* in period *p* (before vs. after the spray), and habitat *h* (forest vs. lawn) was modeled as a log-linear function:

$$\log\left(\mu_{jphl}\right){=\mathrm{intercept}}_{j}{+\mathrm{period}}_{p}{+\mathrm{habitat}}_{h}+{\mathrm{location}}_{l}$$ (1)

The alternative model adds treatment:

$$\log\left(\mu_{jphl}\right){=\mathrm{intercept}}_{j}{+\mathrm{period}}_{p}{+\mathrm{habitat}}_{h}+{\mathrm{location}}_{l}+{\mathrm{treatment}}_{t}$$ (2)

We used Akaike Information Criterion values to compare the fit of the two models. If the model that included treatment had a lower AIC value, then we concluded that treatment significantly affected abundance [49]. Analysis of deviance (anova.manyglm in mvabund) was used to determine the significance of each term in the best-fitting model.

The arthropod communities represented by the bulk samples versus pitfall samples may respond differently to Met52, due to differences in interactions among taxa, mobility, and seasonality. Therefore, we analyzed bulk and pitfall data separately. Within each sample type, two sets of analyses were performed considering the two post-spray samples, because immediate post-spray arthropod responses may have differed from responses several weeks later. The first set of analyses included data from samples taken pre-spray and 1 week post-spray. The second set of analyses included pre-spray samples and the second set of post-spray samples.

The number of observations was not much larger than the number of predictors, preventing estimation of the the correlation matrix across taxa. Therefore, we assumed taxa responded independently. In mvabund, the significance of the test statistic (the likelihood ratio) is evaluated via resampling rows of data, preserving the correlation structure across orders within locations, habitats, and sampling occasions. Therefore, inferences made in mvabund are valid even when taxa exhibit correlated responses [50].

#### Before-After-Control-Impact effects

The observed data were used to calculate the means and standard errors for each period-treatment category. The Before-After-Control-Impact (BACI) effect for each order was calculated as the difference in average abundance, *μ*_*j*_, between Met52 and H_2_O plots, for samples after the spray, minus the difference before the spray: (*μ*_*jhl*,*p = after*, *t = Met52*_ - *μ*_*jhl*,*p = after*, *t = H2O*_)—(*μ*_*jhl*,*p = before*, *t = Met52*_ - *μ*_*jhl*,*p = before*, *t = H2O*_) [51]. BACI standard errors were computed from the set of BACI effect values for each location and habitat.

#### Power analyses

We used bootstrapping to conduct both retrospective and prospective power analyses [52,53] (R code available: S1 Code). The objective of the retrospective power analysis was to determine the percent reduction in abundance that was detectable with 80% power, given the data that we collected. The analysis addressed changes in abundance in the bulk samples and pitfall samples taken pre-treatment and in the two post-treatment sampling occasions. For each randomization run, counts were generated for each observation by sampling with replacement from the set of observed pooled samples. By randomizing at the scale of samples, rather than taxa, this randomization procedure preserved potential correlations in abundance across taxa present in the original dataset. Following these random draws, the values in the Met52 samples, post-treatment, were multiplied by one of a range of reduction factors, from 0.1 to 0.9 in increments of 0.05, representing a range of reductions in abundance. As with analyses previously described for the observed data, two alternative multivariate GLMs were fitted to the randomly generated dataset: a full model with period, habitat, and treatment as predictors (Eq 2), and a nested null model without treatment (Eq 1). If the model including treatment had the lower AIC value, then the effect of Met52 was considered to have been detected for that randomization run and level of reduction in abundance. The randomization and testing procedure was repeated 10,000 times for each reduction level, generating a distribution of AIC values for the two alternative GLMs for each reduction level. If the full model including treatment was the better fit in at least 80% of randomization runs, then the study design was estimated to have 80% power to detect the specified reduction in abundance. We identified the smallest reduction in abundance for which there was at least 80% power to detect this change.

The objective of the prospective power analysis was to determine the sample size that would be needed in a future study to have 80% power to detect either a 25% or a 50% reduction in arthropod abundance due to Met52 treatment, considering the first post-treatment sample. In the context of biocontrol, fifty percent reduction in abundance of a non-target population is a level that has been considered feasible for detection and ecologically meaningful [54–56]. We simulated larger sample sizes by drawing with replacement from the observed data. For bulk samples, we simulated multiplying sample size by a range of factors from one (no change in sample size) to twenty. Given the smaller observed set of pitfall samples, we simulated a range of pitfall samples from 10 to 100 times the observed sample size, in increments of ten. We simulated each scenario of reduction in arthropod abundance and increase in sample size 1,000 times. As with the retrospective bootstrap power analysis, for each randomization run we determined whether there was a significant effect of Met52 based on comparison of AIC values from two alternative GLMs. R Code is available via figshare [57].

## Results

### Bulk samples

The 156 pooled samples contained an estimated 124,983 arthropods, including 89,280 Acari and 25,938 Collembola (extrapolated from subsamples), and 7,008 individuals across 18 other orders. The null model had a better fit to the data (AIC = 6416) than the model including treatment (AIC = 6431, delta AIC = 15), considering samples taken pre-spray and 1 week post-spray (Table 1). Analysis of deviance of the best fitting model indicated significant effects of habitat (likelihood ratio [LR] = 153.8, P = 0.001) and plot location (LR = 332.9, P = 0.003), with no effect detected for period (LR = 23.1, P = 0.35) (S1 Table).

**Table 1:** Comparison of alternative models for abundance of arthropods in bulk samples taken pre-treatment and 1 week post-treatment. The best fitting model included as predictors period, habitat, and location, but not treatment.

Model	Res.Df	Likelihood ratio	P(>LR)	AIC.value	delta.AIC
abundance ~ period + habitat +location	89	NA	NA	6416	0
abundance ~ period + habitat + location + treatment	88	26.5	0.23	6431	15

Considering bulk samples taken pre-spray and 3 weeks post-spray, the null model again had a better fit to the data (AIC = 6795) compared to the model including treatment (AIC = 6826, delta AIC = 31) (Table 2). The best fitting model had significant effects of period (LR = 51.0, P = 0.03), habitat (LR = 187.1, P = 0.001), and plot location (LR = 382.5, P = 0.001) (S2 Table).

**Table 2:** Comparison of alternative models for abundance of arthropods in bulk samples taken pre-treatment and 3 weeks post-treatment. AIC values indicated the best fitting model included effects of period, habitat, and location, but not treatment.

Model	Res.Df	Likelihood ratio	P(>LR)	AIC.value	delta.AIC
abundance ~ period + habitat + location	89	NA	NA	6795	0
abundance ~ period + habitat + location + treatment	88	11.0	0.92	6826	31

Retrospective power analysis indicated that the study had at least 80% power to detect a reduction in arthropod abundance of 50% or greater, considering samples taken 1 week after the spray, and a reduction of 60% or greater, considering samples taken 3 weeks post-treatment. To have at least 80% power to detect a 50% reduction in abundance 1 week post-treatment, three times the current sample size would be needed, while eight times the current sample size would be needed to achieve at least 80% power to detect a 25% reduction in abundance.

**Fig 1:**
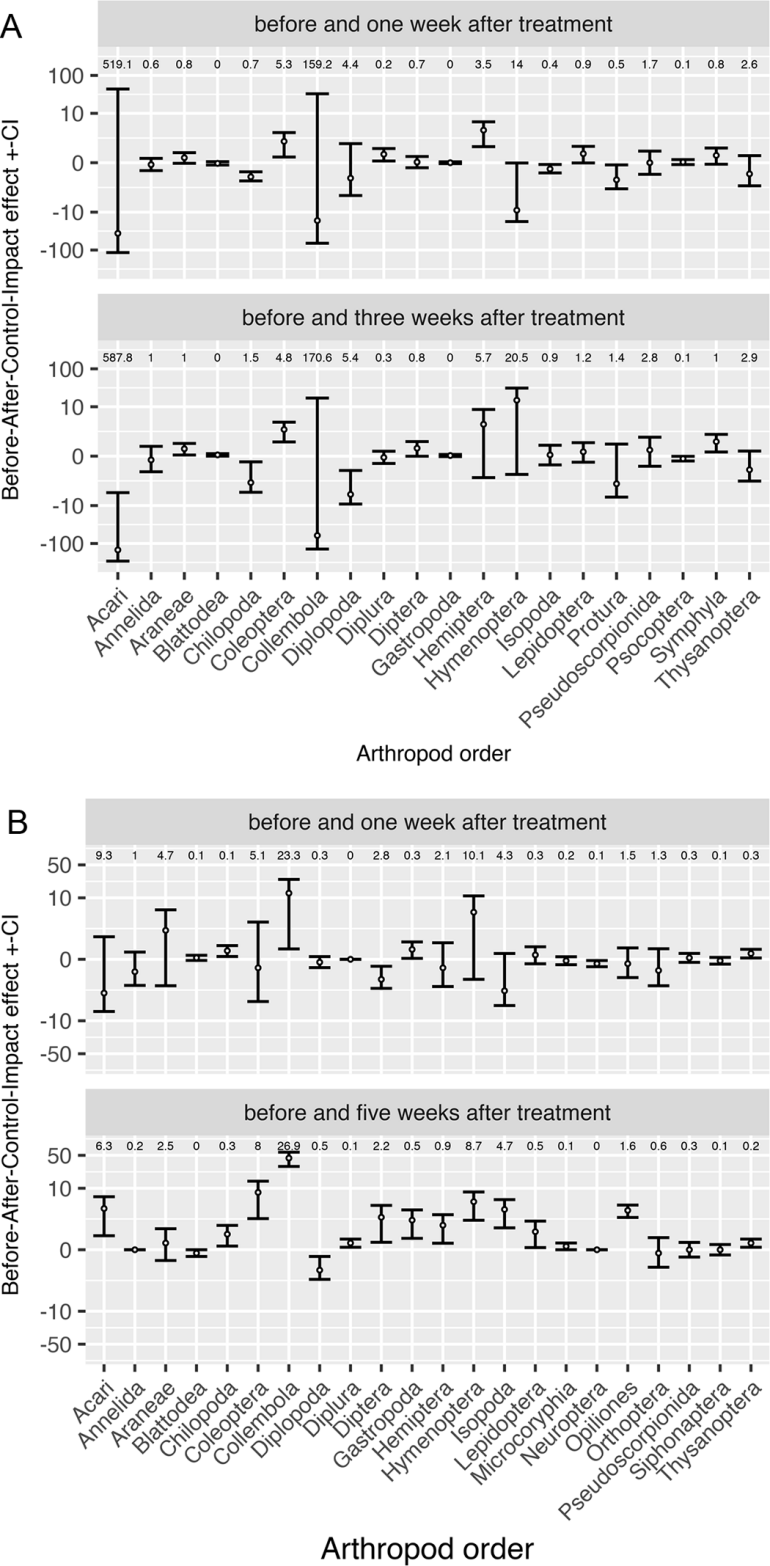
**Before-After-Control-Impact (BACI) effects for bulk samples (A) and pitfall samples (B).** For bulk samples, BACI effects were based on samples taken pre-treatment and 1 week post-treatment (A, top panel) and based on samples taken pre-treatment and 3 weeks post-treatment (A, bottom panel). For pitfall samples, BACI effects were based on samples taken pre-treatment and 1 week-post treatment (B, top panel), and pre-treatment and 5 weeks post-treatment (B, bottom panel). For arthropod order *j*, the BACI effect is: (*μ*_*j*,*p = after*, *t = Met52*_ - *μ*_*j*,*p = after*, *t = H2O*_)—(*μ*_*j*,*p = before*, *t = Met52*_ - *μ*_*j*,*p = before*, *t = H2O*_). Standard errors were computed from BACI effects observed for order *j* at each location and habitat. Values are plotted on an inverse hyperbolic sine scale. Above the BACI for each order is the mean abundance for that order across all period-treatment categories for that sample type (bulk vs. pitfall).

The estimated BACI effects for each order in the bulk samples, for the two before-after comparisons, were generally low, with standard errors almost always encompassing 0 (Fig 1A; S3 Table). Within each order, standard errors for abundance in Met52 and water plots almost always overlapped at each sampling occasion (S1 Fig).

### Pitfall samples

The 68 pooled samples contained 4,276 individuals in 22 orders, the three most abundant orders being Collembola (1,424 specimens), Hymenoptera (634), and Acari (566). The null model provided the best fit to the data (AIC = 2713), compared to the model including treatment as a predictor (AIC = 2717, delta AIC = 4), considering samples collected pre-treatment and 1 week post-treatment (Table 3). For the best-fitting model, there was a significant effect of location (LR = 264.9, P = 0.001), period (LR = 46.2, P = 0.013), but not of habitat (LR = 26.7, P = 0.18) (S4 Table).

**Table 3:** Comparison of alternative models for abundance of arthropods in pitfall samples taken pre-treatment and 1 week post-treatment. AIC values indicated the best-fitting model included effects of period, habitat, and location, but not treatment.

Model	Res.Df	Likelihood ratio	P(>LR)	AIC.value	delta.AIC
abundance ~ period + habitat +location	47	NA	NA	2713	0
abundance ~ period + habitat + location + treatment	46	31.2	0.098	2717	4

For pitfall samples taken pre-spray and five weeks post-spray, the null model was again better supported (AIC = 1841) than the model that included treatment (AIC = 1849, delta AIC = 8) (Table 4). In the best fitting model, there were significant effects of period (LR = 38.9, P = 0.03), habitat (LR = 62.4, P = 0.001), and location (LR = 209.8, P = 0.001), (S5 Table).

**Table 4:** Comparison of alternative models for abundance of arthropods in pitfall samples taken pre-treatment and 5 weeks post-treatment. AIC values indicated the best-fitting model included effects of period, habitat, and location, but not treatment.

Model	Res.Df	Wald test statistic	P(>Wald)	AIC.value	delta.AIC
abundance ~ period + habitat	31	NA	NA	1841	0
abundance ~ period + habitat + treatment	30	27.2	0.19	1849	8

For the pitfall samples taken before and 1 week post-treatment, most of the BACI effects are low, with standard errors that include 0 (Fig 1B, top panel; Supporting Information: S6 Table). For samples taken 5 weeks post-treatment, BACI effects remain low, with about half the orders having positive effects and standard error ranges above 0 (Fig 1B, bottom panel; S6 Table). Order-level abundances followed similar paths over time in the Met52 and control plots (S2 Fig).

Retrospective power analysis indicated 8% power to detect a 90% reduction in abundance for pitfall samples taken 1 week post-spray, and 10% power for samples 5 weeks post-spray. Prospective power analysis indicated that increasing sampling by up to a factor of one hundred would yield a maximum of 7% power to detect 25% reduction in abundance, or maximum 6% power to detect 50% reduction in abundance, considering samples taken 1 week post-treatment.

Data are available from figshare [57].

## Discussion

Met52 is one of a range of biocontrol agents developed for use against vectors for human disease. Exposure to the ticks that transmit tick-borne pathogens in the eastern and central United States is thought to occur peridomestically [11–13], resulting in widespread interest in developing effective, safe methods for controlling ticks in yards [24]. Containing the fungus *Metarhizium brunneum* strain F52, Met52 has shown the potential to control ticks in yards to a comparable degree to that achieved with chemical pesticides [21,58]. It is important to assess whether Met52 has unintended consequences for non-target arthropods that share the ticks’ environment. In the lab, Met52 has had no effect on some non-target taxa, yet increased mortality in others [59]. In the field, the non-target effects of Met52, and other *M*. *brunneum* strains, have been primarily assessed in agricultural settings [34].

The Tick Project (www.tickproject.org) is an ongoing study testing whether TBD can be reduced through neighborhood-scale yard treatment with Met52, by itself or together with bait boxes that apply the acaricide fipronil to small mammals. The Tick Project is the first neighborhood-scale use of Met52. Given the efficacy of Met52 against diverse target taxa, it is plausible that it would negatively impact non-target arthropods. If Met52 caused declines in non-target arthropods, or disruptions in ecosystem functions performed by non-target arthropods, these costs would need to be weighed against the potential tick control benefits of Met52.

This study reports the first field test of the non-target effects of Met52 as applied for tick control in lawn and forest habitats typical of residential yards. Non-target arthropods were sampled, via bulk samples of soil and litter and via pitfalls, before and after spraying plots with Met52 or water (control plots). Multivariate generalized linear models [47] were used to jointly predict the abundances of arthropod orders. Across sample types (bulk, pitfall) and two post-spray sampling occasions, the better fitting models included as predictors location, period, and habitat, but not treatment. Power analysis indicated the study design had at least 80% power to detect reductions in abundance of 50% or greater, considering arthropods in bulk samples taken 1 week post-spray. It is possible that Met52 caused lesser changes in arthropod abundance, which this study was less likely to detect. Considering non-target arthropod communities as a whole, however, the experimental results indicated that use of Met52 in yards is unlikely to have major negative impacts on arthropod populations or communities.

Based on the expected Type I error rate, interpreting the results of unadjusted univariate tests to ~20 taxa is expected to result in 1 taxon exhibiting a significant effect of treatment at the P<0.05 level by chance, even if there is no real treatment effect. On the other hand, with 20 taxa, making adjustments for multiple comparisons reduces the likelihood of detecting changes in abundance that may be ecologically significant but not meet a P<0.05 cutoff. Therefore, possible patterns in the BACI effects are identified but without drawing conclusions about statistical significance.

For Acari and Collembola, the two most abundant taxa in the bulk samples, the BACI effects were negative (Fig 1A), with large standard errors. Negative effects for Acari would be consistent with the effects of Met52 on ticks [60], spider mites [61], and predatory mites [30]. Negative effects for Collembola would be consistent with a study that found increased mortality following exposure to BIPESCO 5 (= F52) [28]. Among less abundant taxa, some appeared to have positive BACI effects (e.g., Hymenoptera, Coleoptera, Hemiptera), with others being negative (Chilopoda, Diplopoda). We do not know whether these possible patterns are ecologically significant.

Considering the pitfall data (Fig 1B), the BACI effect is positive for Acari and Collembola for the samples taken 5 weeks post-treatment. Pitfall samples captured more mobile arthropods, which may have been able to recolonize more rapidly, compared to arthropods in bulk samples. In the lab, BIPESCO 5 attracted collembolans, and one species exhibited no increase in mortality after consuming BIPESCO 5 [28]. It is possible that some collembolans were attracted to, and even benefited from, Met52. Hymenoptera, second-most abundant in the pitfall samples, exhibited positive BACI effects. A positive effect on Hymenoptera would be consistent with a study at found increased abundance of ants in BIPESCO 5 plots [34]. Ants exhibit a range of behavioral and immune defenses against *M*. *brunneum* [62].

Bulk sampling was clearly the more useful sampling method. Power analysis for the pitfall data indicated that the power to detect even a 90% reduction in abundance was approximately equal to the expected Type I error rate. Tripling the current sample size would result in 80% power to detect a 50% change in arthropods in the bulk samples, whereas even increasing sample size 100-fold would not increase power with arthropods sampled by pitfall.

The total area of the 13 treated plots, 832 square meters, was about 0.01% of the 8 square kilometers of the CIES campus. If Met52 caused reductions in abundance of non-target taxa, there was a large surrounding area from which affected taxa could recolonize. Even major reductions in abundance would be unlikely to significantly affect population or community ecology or ecosystem function in the landscape. In The Tick Project, 23–43% of about 100 properties in a contiguous area receive treatment with Met52 (or control) twice each year for four years, beginning in 2017. At this greater scale of Met52 treatment, it is possible that non-target impacts may emerge that were not found in the present study.

## Supporting information

S1 Code R code for retrospective and prospective bootstrap power analysis. (ZIP)

S1 Table Analysis of deviance for the best-fitting model of arthropod abundance in bulk samples, considering data taken pre-treatment and 1 week post-treatment. There was a significant effect of habitat.
(CSV)

S2 Table Analysis of deviance for the best-fitting model of arthropod abundance in bulk samples, considering data taken pre-treatment and 3 weeks post-treatment. There were significant effects of period, habitat, and location.
(CSV)

S3 Table Bulk sample means and BACI effects. Order-level means (standard errors) and Before-After-Control-Impact effects (SEs) for bulk samples.
(CSV)

S4 Table Analysis of deviance for the best-fitting model of arthropod abundance in pitfall samples, considering data taken pre-treatment and 1 week post-treatment. There was a significant effect of period.
(CSV)

S5 Table Analysis of deviance for the best-fitting model of arthropod abundance in pitfall samples, considering data taken pre-treatment and 5 weeks post-treatment. There was a significant effect of period and habitat.
(CSV)

S6 Table Pitfall sample means and BACI effects. Order-level means (standard errors) and Before-After-Control-Impact effects (SEs) for pitfall samples.
(CSV)

S1 Fig Arthropod abundance over time in bulk samples. Mean and standard error abundance for each order and sampling occasion for Met52 and control (H_2_O) plots for bulk sample data.
(PNG)

S2 Fig Arthropod abundance over time in pitfall samples. Mean and standard error abundance for each order and sampling occasion for Met52 and control (H_2_O) plots for pitfall sample data.
(PNG)
