# Supplementary figures and images for: The tick biocontrol agent Metarhizium brunneum (= M. anisopliae) (strain F52) does not reduce non-target arthropods
Source: PLoS One. 2017 Nov 20;12(11):e0187675. doi: 10.1371/journal.pone.0187675 (PMC5695842; doi:10.1371/journal.pone.0187675)

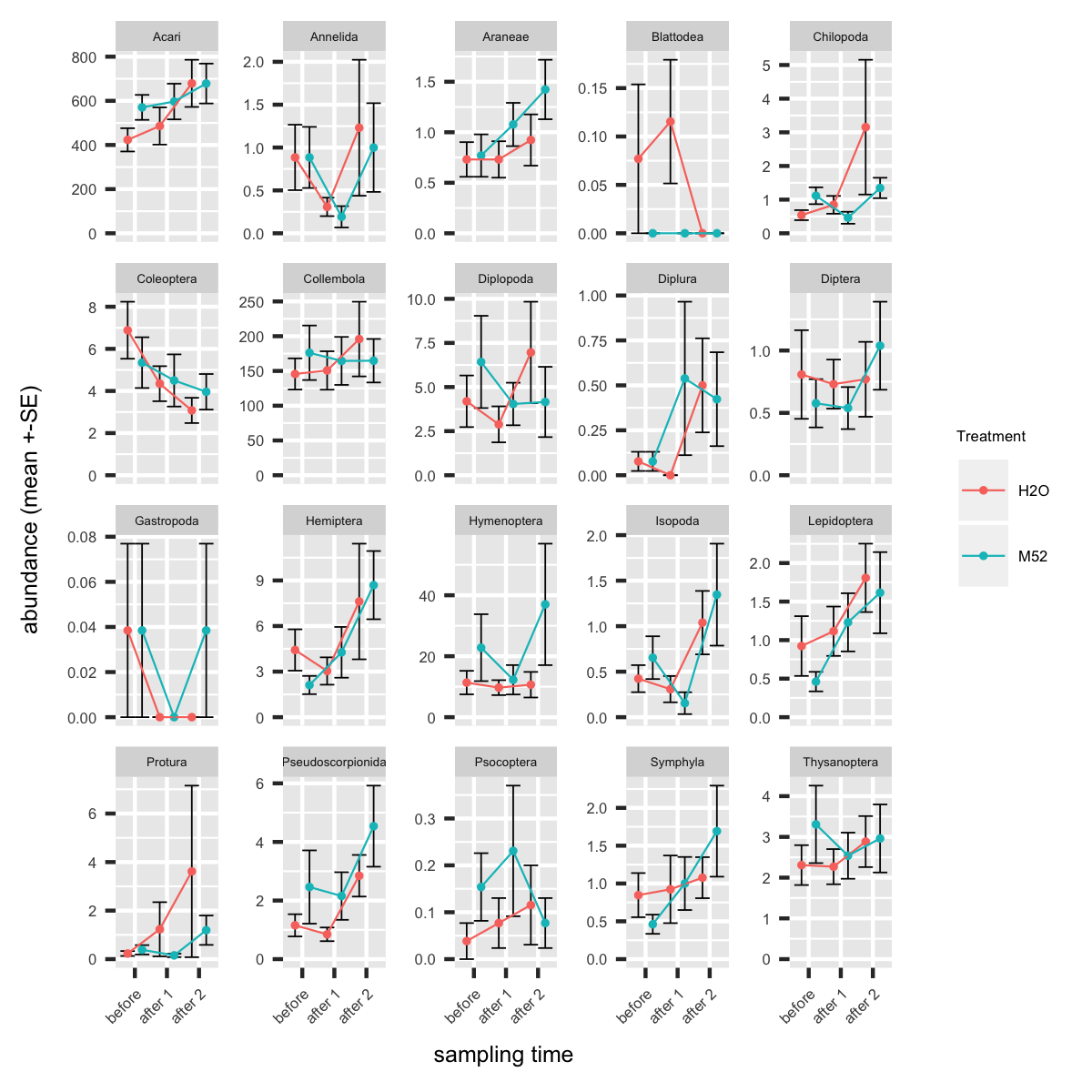

Supplement: S1 Fig — Mean and standard error abundance for each order and sampling occasion for Met52 and control (H2O) plots for bulk sample data. (PNG) [file pone.0187675.s008.png]

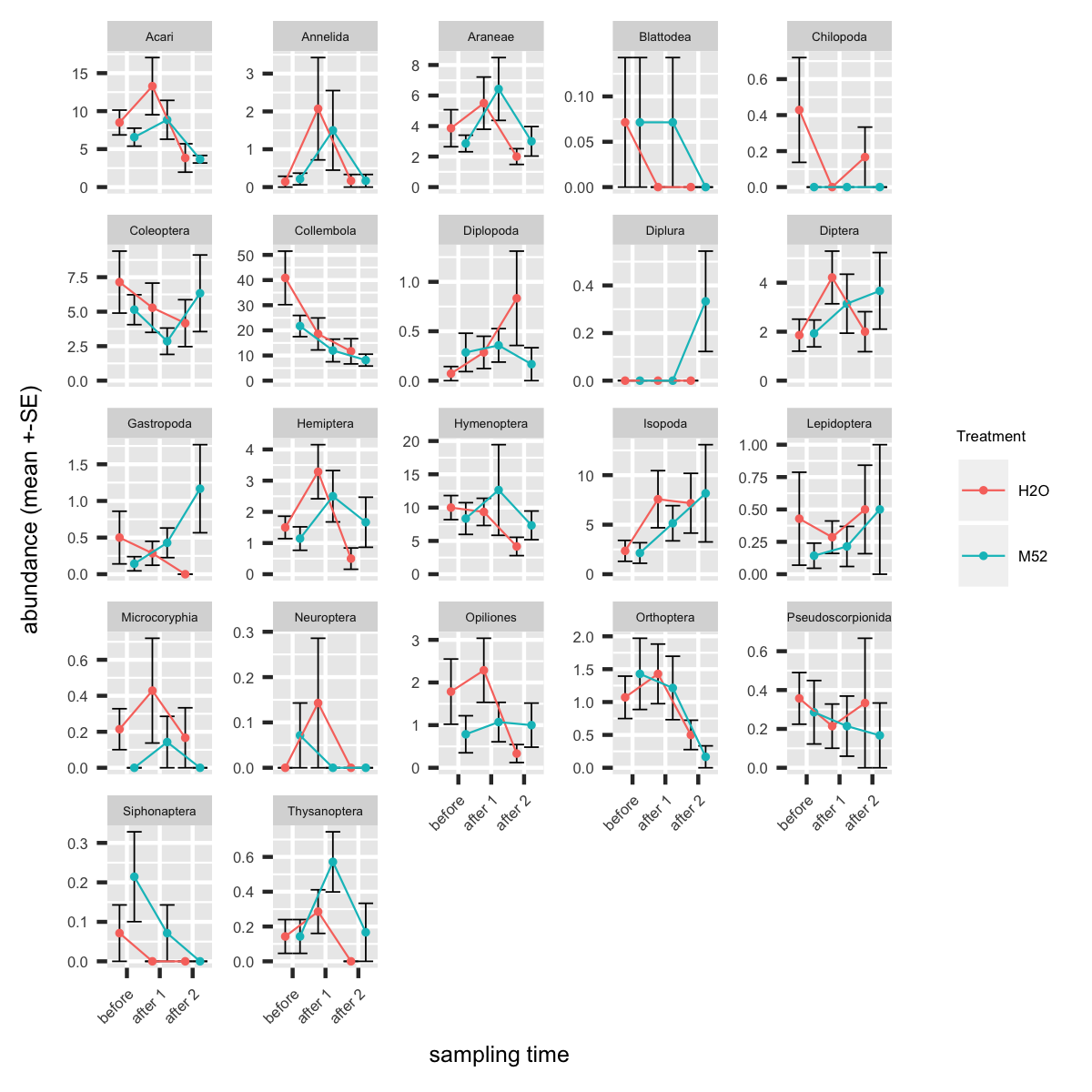

Supplement: S2 Fig — Mean and standard error abundance for each order and sampling occasion for Met52 and control (H2O) plots for pitfall sample data. (PNG) [file pone.0187675.s009.png]
